# Predicting the post-treatment recovery of patients suffering from traumatic brain injury (TBI)

**DOI:** 10.1007/s40708-015-0010-6

**Published:** 2015-02-27

**Authors:** Zaigham Faraz Siddiqui, Georg Krempl, Myra Spiliopoulou, Jose M. Peña, Nuria Paul, Fernando Maestu

**Affiliations:** 1 Research Lab “Knowledge Management and Discovery” (KMD), Faculty of Computer Science, Otto-von-Guericke University Magdeburg, Magdeburg, Germany; 2 CeSViMa Supercomputing and Visualization Center, Technical University of Madrid, Madrid, Spain; 3 Department of Basic Psychology I, Complutense University of Madrid, Madrid, Spain; 4 Center for Biomedical Technology, Complutense and Technical University of Madrid, Madrid, Spain

**Keywords:** Traumatic brain injury, Label prediction, Evolution modelling, Clustering, MSC code1, MSC code2, more

## Abstract

Predicting the evolution of individuals is a rather new mining task with applications in medicine. Medical researchers are interested in the progression of a disease and/or how do patients evolve or recover when they are subjected to some treatment. In this study, we investigate the problem of patients’ evolution on the basis of medical tests before and after treatment after brain trauma: we want to understand to what extend a patient *can* become similar to a healthy participant. We face two challenges. First, we have less information on healthy participants than on the patients. Second, the values of the medical tests for patients, even after treatment started, remain well-separated from those of healthy people; this is typical for neurodegenerative diseases, but also for further brain impairments. Our approach encompasses methods for modelling patient evolution and for predicting the health improvement of different patients’ subpopulations, i.e. prediction of label if they recovered or not. We test our approach on a cohort of patients treated after brain trauma and a corresponding cohort of controls.

## Introduction

In the recent years, methods from machine learning and data mining are increasingly being used in the epidemiological and clinical domains. These methods help the clinicians in studying the causes, effects and progression of diseases and the treatments as well. In the context of brain-related degenerative diseases, e.g. traumatic brain injury and mild cognitive impairment, medical researchers want to analyse/monitor the patients suffering from such disease as they evolve over time. In particular, they would like to answer questions like: have the patients reached a similar state like that of healthy people? or given a patient’s current state what is the most suitable treatment regime that can be recommended to him? or how likely is it for a certain patient to recover from the disease? In order to provide answers to these questions, we propose mining methods that learn evolutionary predictive models over the evolving cohort of patients. These methods determine (1) if the patients have achieved a state as that of healthy people by juxtaposing them to a cohort of controls, and (2) given a patient’s current state, will he show recovery after he has been prescribed a treatment regime or plan.

The study of patient evolution on the basis of timestamped clinical data has been largely influenced by the seminal work of Cox [1] on censored failure times and age-specific failure rates. As pointed out by Fitzmaurice et al. [2], the work of Cox [1] “...was followed by a rich and important body of work that established the conceptual basis for the modern *survival analysis*” [2]. Survival analysis is not applicable to this problem, because there is neither a well-defined target event, nor explicit timepoints to guide the learner. Although there is a control population to juxtapose the patients to, there are no target values to predict, because the assessments of the controls are very different from those of the patients. To acquire the labels for the patients, we rely on the recommendations of the clinical experts. We present here a method that learns an evolutionary model from unsupervised data and can also incorporate the labels for supervised evolutionary prediction.

Hospitals in recent years have started to maintain elaborate electronic health records. These store not only the condition or the state a certain patient is experiencing (for example, blood pressure pulse rate, sugar level, etc.) but also keeps track of the medications, their impact and side effects. An important challenge with respect to the impact of a treatment emerges when the desirable target state is not well defined: if clinical data show that patients after treatment are in a different state than before treatment, but they do not exhibit the abilities of a comparable healthy population (controls), what can then be concluded about the impact of treatment? We propose a method that predicts how a treatment improves the state of brain trauma patients, although there is no well-defined target state and the control population exhibits features (values in medical tests) that patients cannot reach.

We study recordings of patient and control cohorts over a certain time horizon. Longitudinal analysis of cohorts is an established and a mature field of research in statistical domain. Focus of the earliest studies in the longitudinal analysis stemmed from the studies on morbidity and mortality [2].

The contributions of our approach are as follows. We model the evolution of subpopulations of patients, for whom only two moments are available, whereby these two moments are not defined as timestamps.^1^ We use this model to compute a future/target state for each patient and also the recovery labels based on clinical recommendations. We show that the projected target state of patients allows a reasonable comparison to a control population, the recordings of which are very different from the patient recordings.

## Related work

Data mining methods are only recently deployed for analysis and prognosis of brain pathologies or injury conditions. The authors of [3] use different methods (e.g. decision trees, multilayer perceptron and general regression neural networks) to analyse data from neuropsychological tests (concerning attention, memory and executive function tests) from 250 subjects before and after a cognitive treatment instrumented by a cognitive tele-rehabilitation platform. Their objective is to predict the expected outcome based on the cognitive affectation profile and the performance on the rehabilitation tasks. Our objective is not the prediction of a well-defined outcome, but rather of the future similarity between treated patients and a population of healthy people.

In [4], the authors present an artificial neural network model that predicts in-hospital survival following traumatic brain injury according to 11 clinical inputs. A similar approach was taken by Shi et al [5], who also consider neural networks and logistic regression, but rather study recovery from brain surgery. An early discussion of methods for prediction of recovery from brain injury, including short-term evolution of patients, can be found in [6]. The effect of cognitive therapies along longer periods (6 months to 1 year) is studied in [7, 8]. Brown et al. learn decision trees on variables that include physical examinations and indices measuring injury severity, as well as gender, age and years of education [7]. Rovlias and Kotsou further consider pathological markers (hyperglycemia and leukocytosis) and the output of computer tomography, and learn CART trees [8]. Our study is different from the aforementioned ones, because we do not learn a model on patient recovery (we do not have recovery data), but rather study the evolution of the patients *towards a control population*.

There are studies [9–13] that track the responses to cognitive-behavioural treatments for brain-related disorder, e.g. post-traumatic disorder, mild cognitive impairment and traumatic brain injury. These studies aim towards finding the response groups based on their developmental trajectories. Methods include group-based trajectory modelling [10, 13] and growth mixture modelling (GMM) [11, 12]. In [13], the method learns developmental trajectories of groups with distinct cognitive change patterns; it uses a cohort of MCI patients. In [12], the authors study the progress of the PTSD (post-traumatic stress disorder) patients on two different therapeutic protocols. Their aims were to identify distinct trajectories of treatment response and to test whether pre-treatment markers predict assignment to those trajectories.

Close to our work are the methods of Tucker et al. [14] and Li et al. [15], who predict the progression of glaucoma from cross-sectional data (rather than longitudinal data). The methods learn temporal models on trajectories. A trajectory is built by fitting so-called “partial paths” upon the cross-sectional data: path construction involves selecting one healthy individual and one patient, labelling them as start and end and then re-ordering the remaining cross-sectional instances based on the shortest path from start to end. Our approach shares with [15, 14] the need to construct a trajectory of evolution. In principle, we could construct a “partial path” by combining the recordings of the controls and the recordings of the patients during treatment. But this would imply ignoring part of the already available temporal information (pre-treatment data). Moreover, the Trauma Brain Injury dataset of [16], which we use, shows that the control individuals are too different from the patients: this might lead to overlong and unrealistic partial paths. Thus, we rather build a single, projected *moment*, using data before and after the begin of treatment, and we do not involve the recordings of the controls in our learning process.

A separate thread of work models and monitors how subpopulations (clusters) evolve over time. The framework MONIC [17] encompasses a set of ’transitions’ that a cluster may experience, a set of measures and a cluster comparison mechanism that assesses whether a cluster observed at some timepoint has survived, disappeared, merged or become split at the next timepoint. Later frameworks [18, 19] build upon MONIC to explain evolution: they model the clusters and their transitions as nodes, resp. edges of an *evolution* graph. In [20], we build upon [19] to learn a Mixture of Markov chains that capture the evolution of different subpopulations. We take up the idea of subpopulations here, but our goal is to predict rather than model the evolution of the subpopulations.

There are also studies concentrating on how individual objects evolve over time. Gaffney and Smith [21] model the evolution of an object as a trajectory and cluster together objects that evolve similarly. Krempl et al. [22] extend [21] into the online algorithm TRACER that discovers and adapts the clusters as new observations of existing objects arrive and new objects appear.

## Label prediction for evolving objects 

### Material

The traumatic brain injury dataset (TBI) contains assessments on cognitive tests for 15 patients with brain injury and for 14 controls [16]. These tests are recorded once for the controls and twice for the patients—at moments $t_{\rm pre}$ and $t_{\rm post}$. The cognitive tests are listed in Table 1 with their acronyms;^2^ a detailed presentation can be found in [16].

**Table 1:** Acronyms and description of cognitive tests from the TBI dataset presented in [16]

Name	Description
TMT-B	Train making test-part B: measures cognitive flexibility (frontal lobe function)
BTA	Brief test of attention (total score)
WCST-NC	Wisconsin card shorting test: percentage total score of conceptual level (number of categories correctly achieved); also measures cognitive flexibility
WCST-RP	Wisconsin card shorting test: # preservative responses (represent error)
FAS	Phonetic fluency test which uses as cues letters F, A, and S as the initial letters for the patients to start the production of words
ICP	Measure a subject’s ability to perform daily activities, and awareness of the disease
CIV	Verbal intelligent quotient (VIQ): measures ability to handle verbal material
CIM	Performance IQ (PIQ): measures ability to handle visio-spatial / non-verbal material
CV	Verbal comprehension index (VCI)
MT	Working memory (WM): measures the subject’s ability to maintain information in short-term memory and recall it
OP	Perceptual organization (PO)
VP	Processing speed index (PSI)
IAC	Attention/concentration index (ACI)
IMG	General memory index (GMI)
IRD	Delayed recall index (DRI)

### Learning a ground truth for the TBI dataset

The data in the TBI dataset are not labelled. For the two timepoints (i.e. $t_{\rm pre}$ and $t_{\rm post}$), we are only provided the scores on how did each of the patient fare wrt. different cognitive tests. In order to compute the labels of the patient after they had undergone treatment, i.e. for timepoint $t_{\rm pre}$, we use the method presented in the following subsection.

*Ground truth*: The opinion of the medical experts suggests that if the computed difference between pre-treatment and post-treatment values of the individual is high, it is more likely that the individual has recovered from traumatic injury. For our experiments, these extracted labels also serve as the ground truth. Our ground truth estimation method uses a similar approach but incorporates additional information. The method is outlined in the following.

**Fig. 1:**
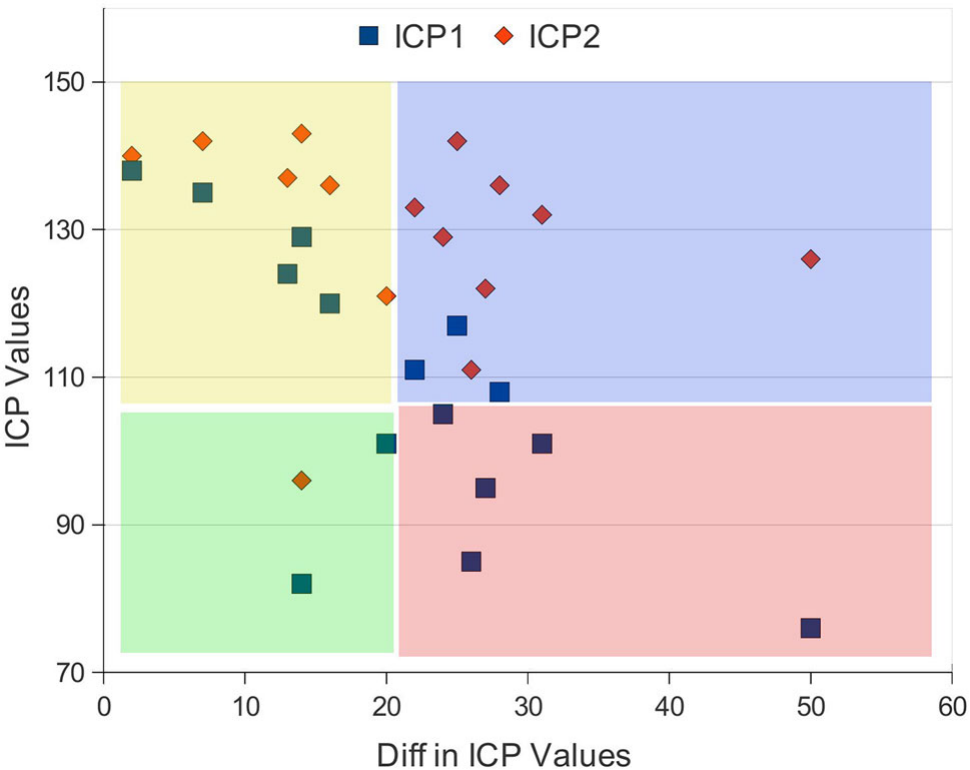
Plot of differences in the ICP values from $t_{\rm pre}$ and $t_{\rm post}$ (x-axis) against *ICP values* (y-axis).* Squares* represent the values for $t_{\rm pre}$, while* rhombuses* represent the values from $t_{\rm post}$. Patients can be separated into 4 classes based on the difference and the ICP values from $t_{\rm pre}$, i.e. Class_1 = [low diff, low ICP1] (*green region*), Class_2 = [high diff, low ICP1] (*red region*), Class_3 = [low diff, high ICP1] (*yellow region*), and Class_4 = [high diff, high ICP1] (*blue region*). (Color figure online)

1. Compute the difference between the values recorded for the variable ICP or WNC during the pre-treatment phase and post-treatment phase for each patient, separately.^3^
2. Plot the pre-treatment values of the used variable (i.e. the one which has been used in step 1) against the computed difference of this variables from the pre-treatment phase to post-treatment phase. We depict an example plot in Fig. 1.
3. Patients can be separated into a number of classes based on regions they fall in within the plot. In Fig. 1, we depict the regions.

### Predicting the recovery of patients

In this section, we present our evolutionary label prediction method EvoLabelPred. This method is based on the unsupervised instance prediction method EvolutionPred of Siddiqui et al. [23]. EvoLabelPred takes as input a labelled longitudinal dataset of individuals. It learns a clustering model over the individual timepoints (i.e. $t_{\rm pre}$ and $t_{\rm post }$), and then learns a cluster-based transition model, the “cluster evolution graph”, by discovering transitions or relationships between the clusters across timepoints. EvoLabelPred uses this cluster transition model to predict the labels of the individuals. In the next section, we first describe learning of transition model and then explain how it is used for predicting the labels of the individuals. A list of used symbols is given in Table 2.

**Table 2:** List of used symbols and notations

Symbol	Description
$t_{\rm pre }$	Timepoint before the start of the treatment
$t_{\rm post }$	Timepoint after the end of the treatment
${\mathcal {X}}$	Set of individuals. The cardinality of the set is* n*
$x_{\rm pre }$	Instance of a patient at timepoint $t_{\rm pre }$
$l_{\rm pre }$	Label of a patient at timepoint $t_{\rm pre }$
$\zeta _{\rm pre }$	Clustering model learned over the instances of individuals from $t_{\rm pre }$
$\zeta _{\rm post }$	Clustering model learned over the instances of individuals from $t_{\rm post }$
$c \in \zeta$	A cluster of individuals from the model $\zeta$
${\mathcal {G}}$	A cluster transition graph learned over clustering $\zeta_{\rm pre }$ and $\zeta_{\rm post }$

#### Bootstrap sampling

EvoLabelPred learns the prediction model from the set of patients ${\mathcal {X}}$. Since the cardinality of ${\mathcal {X}}$ is small (as is the case for many cohort datasets), we learn an ensemble of models by performing bootstrap sampling over ${\mathcal {X}}$. The bootstrap sampling is done *without replacement*, and subsequent instances of each out-of-sample patient (i.e. $x_{\rm pre }, x_{\rm post }$) are removed from both $t_{\rm pre }$ and $t_{\rm post }$.

#### Building a cluster evolution graph

The cluster evolution graph ${\mathcal {G}}$ is learned over each bootstrap sample. Before ${\mathcal {G}}$ can be learned, EvoLabelPred first learns clustering models $\zeta _{\rm pre }$ and $\zeta _{\rm post }$, over the instances of patients from $t_{\rm pre }$ and $t_{\rm post }$, respectively. We apply K-Means over the instantiations at each moment $t$, and build a set of clusters $\zeta _t$.

For learning ${\mathcal {G}}$, we use concepts similar to MONIC [17] and FingerPrint [24] to identify cluster transitions from $t_{\rm pre }$ to $t_{\rm post }$. For each pair of clusters $c\in \zeta _{\rm pre }$ and $c'\in \zeta _{\rm post }$, we compute the extend to which they contain the instances from the same patients. We define their intersection as

$$c\cap {}c'=\{x\in {\mathcal {X}}| x_{\rm pre }\in {}c \wedge x_{\rm post }\in {}c'\},$$

and their union as

$$c\cup {}c'=\{x\in {\mathcal {X}}| x_{\rm pre }\in {}c \vee x_{\rm post }\in {}c'\}.$$

If $c\cap {}c'\ne \emptyset$, we draw an edge $(c,c')$ and assign to it the weight $w_{(c,c')}$

$$w_{(c,c')}=\frac{|c\cap {}c'|}{|c\cup {}c'|}.$$

The learned transition graph ${\mathcal {G}}$ is a directed graph, and all the edges originating from a cluster $c$ sum up to 1, i.e. $\sum _{c_o}w_{(c,c_o)} = 1$.

We define

1 $${ first }\_{ match }(c)={\mathrm {argmax}}_{c'\in \zeta _{\rm post }}w(c,c'),$$

i.e. the *first_match* of a pre-treatment cluster* c* is the post-treatment cluster with the highest weight among the clusters linked to* c*.

**Fig. 2:**
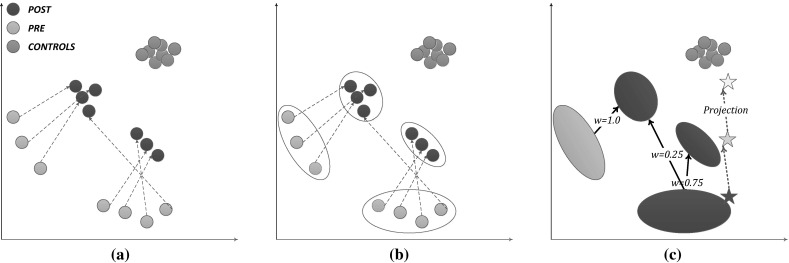
Clustering, Evolution graph and Soft projection with EvolutionPred: in (**a**) the nodes are patient instantiations at $t_{\rm pre }$ (*yellow*) and $t_{\rm post }$ (aubergine), instantiations of the same individuals are connected with* dashed arrows*; additionally, we also show the controls (*green*); (**b**) clustering is performed at each moment (i.e. $t_{\rm pre }$ and $t_{\rm post }$), showing that not all individuals of a pre-treatment cluster evolve similarly; in (**c**) the evolution graph is built by linking pre- and post-treatment clusters that share some individuals; the weights of the graph edges are used to compute the soft projection of the instance denoted by the* red star*. (Color figure online)

In Fig. 2(a), we show the instantiations of example individuals at timepoints $t_{\rm pre }$ (yellow) and $t_{\rm post }$ (aubergine); the corresponding clusters are in Fig. 2(b); the transition arrows along with the transition weights are shown in Fig. 2(c). The yellow star indicates the “projection” of the individual marked as a red star; projections are explained hereafter.

#### Projecting patients into the future

Let $x\in {\mathcal {X}}$ be a patient, $c\in \zeta _{\rm pre }$ be the cluster containing $x_{\rm pre }$, of ${\mathcal {X}}$ and $c_{fm}$ be the ${ first_{match} }(c)$ as of Eq. 1.

*Hard projection:* We define the *hard projection* of $x$ from $t_{\rm pre }$ to $t_{proj }$ as the instantiation of $x$ such that the value of each $a\in {A}$ is determined by the values in $x_{\rm pre }$ and in $\widehat{c},\widehat{c_{fm}}$:^4^

2 $${ projH }(x,t_{\rm pre },t_{\rm post })= x_{\rm pre } + \left( \widehat{c_{fm}} - \widehat{c}\right). $$

The projection is done for each attribute $a \in A$.

*Soft projection*: We define the *soft projection* of* x* from $t_{\rm pre }$ to $t_{proj }$ as an instantiation, the values of which are influenced by all clusters in $\zeta _{\rm post }$ that are linked to* c*:

3 $$ { projS }(x,t_{\rm pre },t_{\rm post })=x_{\rm pre } + \sum _{c'\in \zeta _{\rm post }}\Big( \widehat{c'} -\widehat{c}\Big) \cdot {}w_{c,c'}. $$

The projection is again done for each attribute $a \in A$. Here, $w_{c,c'}$ is the weight of a transition edge.

Hence, we learn models $\zeta _{\rm pre }$ and $\zeta _{\rm post }$ on some individuals and then assess the projection location of other (or the same) individuals. In Fig. 2(c), we show the *soft projection* of an individual (red star): the projected position is outside both post-treatment clusters, since the individual is located at the rim of the pre-treatment cluster.

#### Predicting patient recovery

To predict the next label of a patient, we use a prediction method EvoLabelPred that uses cluster transition ${\mathcal {G}}$ and learns conditional probabilities over each cluster. The method is depicted in Algorithm 2 and we explain it in the following.

*Learning conditional probabilities*: For each cluster $c\in \zeta _{\rm pre }$, iterate over all the patients that are members of $c$. For each label $l$ in $t_{\rm pre }$ and each label $l'$ in $t_{\rm post },$ we compute the occurrences of patients who undergo label transition $l \rightarrow l'$. We compute the conditional probability using the following:

4 $$ P(l'|l) = \frac{ {\mathrm {count}} (\forall x | l_{\rm pre} \ is \ l \wedge l_{\rm post} \ is \ l')}{{\mathrm {count}} (\forall x | l_{\rm pre} \ is \ l) }. $$

*Label prediction*: We define the *label prediction* $\hat{l}_{\rm post }$ of $x$ from $t_{\rm pre }$ to $t_{\rm post }$ as the label that is computed using the conditional probability model inside each cluster $c \in \zeta _{\rm pre }$. Let $c$ be the cluster in $\zeta _{\rm pre }$ that is closest to $x$, the label can then be computed using the following:

5 $$ { predCL }(x,l_{\rm pre }) = {\mathrm {argmax}}_{l' \in c} P(l'|l_{\rm pre }).$$

## Evaluation

In this section, we evaluate our methods on predicting the recovery of patients with traumatic brain injury. Details about the dataset have already been presented in Sect. 3.1. Here, we describe our evaluation framework.

### Evaluation settings and framework

We have presented two methods, i.e. one for projecting the patients into future given his current state, EvolutionPred, and the other for predicting the recovery of the patients, EvoLabelPred, given his current state and current label, e.g. at $t_{\rm pre}$.

#### Framework for EvolutionPred

To evaluate the performance of the projections from EvolutionPred, we are inspired by the mean absolute scaled error (MASE) [25], which was originally designed to alleviate the scaling effects of mean absolute error(MAE). To define our variation of MASE, we assume an arbitrary set of moments ${\mathcal {T}}=\{t_1,t_2,\ldots ,t_n\}$. For an individual $x$, we define the MASE of the last instantiation $x_n$ as

$$ MASE(x)={d(x_{proj },x_n)}/ {\frac{1}{n-1}\sum _{i=2}^{n-1}d(x_i,x_{i-1})},$$

where $d()$ is the function computing the distance between two consecutive instantiations of the same individual* x*. This function normalizes the error of EvolutionPred at the last moment $t_n$ (nominator) to the error of a naive method (denominator), which predicts that the next instantiation of* x* will be the same as the previous (truly observed) one. If the average distance between consecutive instantiations is smaller than the distance between the last instantiation and its projection, then MASE is larger than 1. Obviously, smaller values are better.

We further compute the $Hits()$, i.e the number of times the correct cluster is predicted for a patient* x*. Assume that instantiation $x_{\rm pre}$ belongs to cluster $c_{\rm pre}$ and let $c_{proj }$ denote the ${ first_{match} }(c_{\rm pre})$ (cf. Eq. 1) at the projection moment $t_{proj }$. We set $Hits(x)=1$, if $c_{proj }$ is same as $c_{\rm post}$ (i.e. cluster closest to $x_{\rm post}$), otherwise $Hits(x)=0$. Higher values are better.

For model purity, we compute the entropy of a cluster* c* towards a set of classes $\xi$, where the entropy is minimal if all members of* c* belong to the same class, and maximal if the members are equally distributed among the classes. We aggregate this to an entropy value for the whole set of clusters $\zeta$, $entropy(\zeta ,\xi )$.

In general, lower entropy values are better. However, the labels used by the EvolutionPred are Control and Patient: if a clustering cannot separate well between patient instantiations and controls, this means that the patient instantiations (which are the result of the projection done by EvolutionPred) have become very similar to the controls. Hence, high entropy values are better.

For learning evolutionary prediction model, we use a bootstrap sampling [26] with a sample size of 85 % and 10,000 replications. Model validation is done with the help of out-of-sample data. For clustering the union of projected instances and the controls, we use K-Means clustering. We use bootstrap sampling with a sample size of 75 % and 1,000 replications, and vary $K=2, \ldots 8$.

#### Framework for EvoLabelPred

In order to evaluate EvoLabelPred, we use accuracy to assess the quality of computed labels towards the ground truth that we established in Sect. 3.2. Additionally, we will vary the parameter for the number of the subgroups, i.e.* K* = 3, 4.

To learn an evolutionary label prediction model, we use a bootstrap sampling [26] with a sample size of 85 % and 5,000 replications. Sampling is done *without replacement*, i.e. duplicates are not allowed. Model is validated on the objects that are outside of the sample.

### Evaluation results

#### Evaluating evolutionary projection

**Fig. 3:**
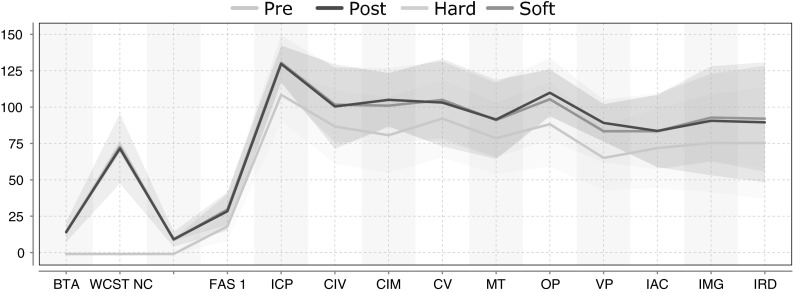
Variance plots for patient projections, where $t_{proj }$ is set to predict the (already known) instances at $t_{\rm post }$: the* solid lines* represent the mean values of the true patient instantiations at moment $t_{\rm pre }$, $t_{\rm post }$ and of the projected patient instantiations, while the surrounding regions (*same colour* as the* solid line*) represent the variance of the instantiations; the two projections overlap almost completely with the true distribution at $t_{\rm post }$, both with respect to the line of the mean and to the region of the variance. (Color figure online)

*Validation of the projection from* $t_{\rm pre }$ *to* $t_{\rm post }$: In the first experiment, we project the patient instantiations from $t_{\rm pre }$ to $t_{\rm post }$. Since the true instantiations at $t_{\rm post }$ are known, we use these projections to validate EvolutionPred, whereupon evaluation is done with the MASE and Hits measures (cf. Sect. 4.1). Figure 3 depicts the hard and soft projections of the pre-treatment patient instantiations, while Table 3 depicts the MASE and Hits values for each patient separately. We perform 10,000 runs and average the values per run.

In Fig. 3, we can see that the hard projection (yellow) and soft projection (green) behave very similarly. Both predict the patient instantiations at $t_{\rm post }$ very well: the mean values for the projected patient instantiations are almost identical to the true instantiations, and the shaded regions (capturing the variance around the mean) overlap with the variance of the true values almost completely.

**Table 3:**
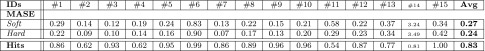
Hard and soft projection of patients from $t_{\rm pre }$ towards $t_{\rm post }$, with MASE and Hits per patient: low MASE is better, values larger than 1 are poor; high Hits are better, 1.0 is best; averages over all patients after excluding outlier patient #14

The first row of Table 3 enumerates the 15 patients in the TBI dataset, and the subsequent rows show the MASE values for the hard, respectively, the soft projection. The last row shows the Hits value per patient. The last column averages the MASE and Hits values over all but one patient: patient #14 is excluded from the computation, because prior inspection revealed that this patient is an outlier, for whom few assessments are available. All other patients exhibit low MASE values (lower is better), indicating that our projection mechanisms predict well the patient assessments at $t_{\rm post }$.

**Fig. 4:**
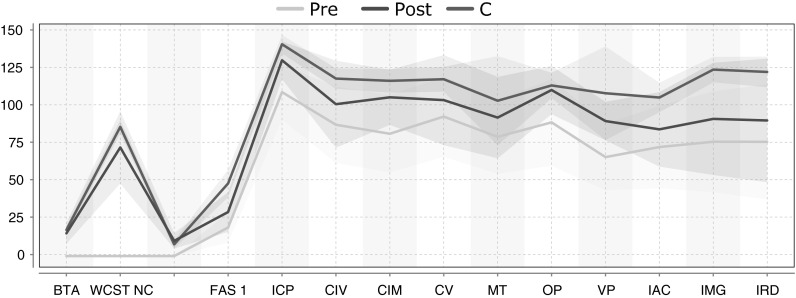
Average assessment values and variance regions for controls and for patients before (Pre) and after treatment start (Post) for 16 variables: despite some overlaps, lines and regions of patients are mostly distinct from those of the controls. (Color figure online)

*Projection from* $t_{\rm post }$ *to the future* $t_{\rm proj }$: In the second experiment, our EvolutionPred projects the patients after treatment start towards a future moment $t_{proj }$, which corresponds to an ideal final set of assessments that the patient might ultimately reach through continuation of the treatment. We do not have a ground truth to evaluate the quality of our projections. Rather, we use a juxtaposition of patients and controls, as depicted in Fig. 4. We show the averages of values per population through a solid line, around which we expand to the variance of values for each variable. The cyan line and surrounding cyan-shaded region stand for the moment

$t_{\rm pre }$, denoted as “Pre” in the legend; the blue line and region stand for the moment $t_{\rm post }$ (“Post”), while the “Controls” are marked by the red line and red-shaded region. Except for Gender and Age, for which controls have been intentionally chosen to be similar to the patients, patients differ from controls. Even where we see overlap between the red area and the cyan (Pre) or the blue (Post) area of the patients, as for assessments CIM and CV, we also see that the average values are different.

**Fig. 5:**
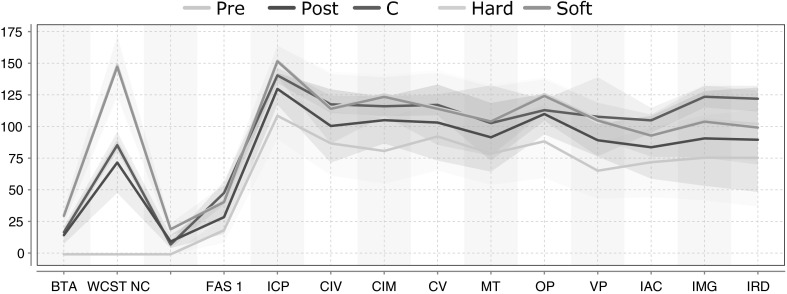
Average assessment values and variance regions for controls and for patients before (Pre) and after treatment start (Post), and as result of Hard (*yellow*) and Soft (*green*) projection: the projected patient assessments are closer to the controls. (Color figure online)

Figure 5 shows the same lines and areas for assessments before and after treatment start (Pre:cyan, Post:blue) as shown in reference Fig. 4, but also the projected assessment values (Proj: green/yellow). These projected assessments are closer to the controls, indicating that at least for some of the assessments (FAS1, ICP, CIM, CV, MT, VP), treatment continuation may lead asymptotically to similar values as for the controls.

**Fig. 6:**
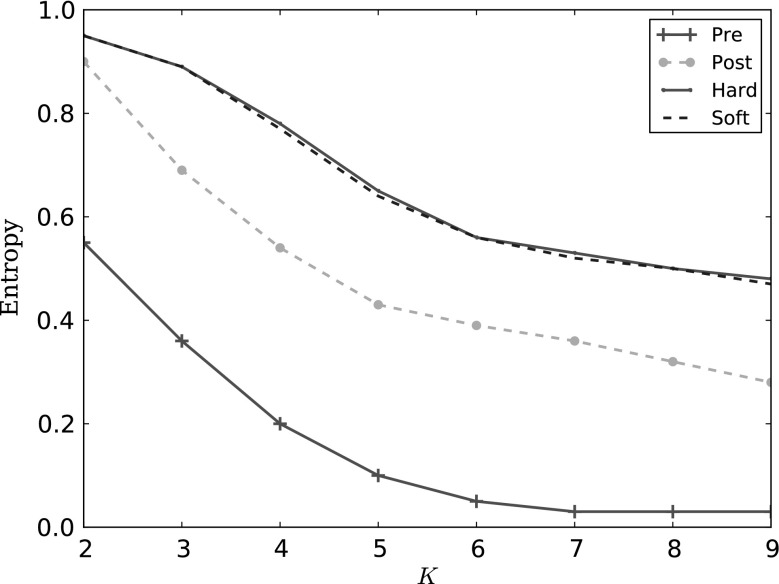
Controls clustered with the patients before treatment (Pre:* red*), after treatment start (Post:* yellow*), with the Hard projection (*green*) and the Soft one (*blue dashed*): entropy drops as the number of clusters increases, but has higher (better) values for the projected instantiations, indicating that these are closer to the controls

*Clustering patients with controls*: We investigate whether the patients can be separated from the control population through clustering. We skip the assessments TMT-B, BTA, WCST-NC and WCST-RP, which have been recorded only for some patients. We cluster the controls with the patient instantiations before treatment (Pre: red line), after treatment start (Post: yellow line), with the Hard projected instantiations (green line) and with the Soft projection (blue dashed line). We use bootstrapping with a sample size of 75 % with 1,000 replications. In Fig. 6, we show the entropy while we vary the number of clusters* K*. *Higher* values are better, because they mean that the clustering cannot separate controls from patients. High values are achieved only for the projected instantiations.

In Fig. 6, the entropy values are very high for the clusters containing controls together with projected patients, whereby soft projection and hard projection behave identically. The high values mean that the clustering algorithm cannot separate between projected patients and controls on similarity; the instances are too similar. This should be contrasted with the clusters containing controls and patients before treatment (red line): entropy is low and drops fast as the number of clusters increases, indicating that patients before treatment are similar to each other and dissimilar to controls. After the treatment starts, the separation between patients and controls on similarity (yellow line) is less easy, but an increase in the number of clusters leads to fair separation. In contrast, projected patients are similar to controls, even when the number of clusters increases: the small clusters contains still both controls and patients.

#### Evaluating evolutionary label prediction

**Table 4:** Label prediction accuracies of each patient for EvoLabelPred with *GroundTruth* based on *ICP* attribute

ID	EvoLabelPred	
	*K* = 3	*K* = 4
#1	0.00	0.00
#2	0.93	0.91
#3	0.41	0.23
#4	1.00	0.70
#5	0.00	0.01
#6	0.03	0.01
#7	0.00	0.00
#8	0.91	0.87
#9	0.05	0.01
#10	0.95	0.87
#11	1.00	1.00
#12	0.89	1.00
#13	0.09	0.09
#14	0.50	0.16
#15	0.95	0.89

We present the results from the label prediction experiments on TBI dataset in Table 4. In the experiment, we first learned the evolutionary model using EvoLabelPred with $K=3, 4$ and then utilized the conditional probabilities-based label prediction (cf. Sect. 3.3.4) within each individual cluster to predict the labels for the out-of-sample patients. The accuracy of label prediction for the label learned from ICP variable is very low: the method is able to achieve a very high accuracy for some of the patients, but if fails completely for other patients.

**Fig. 7:**
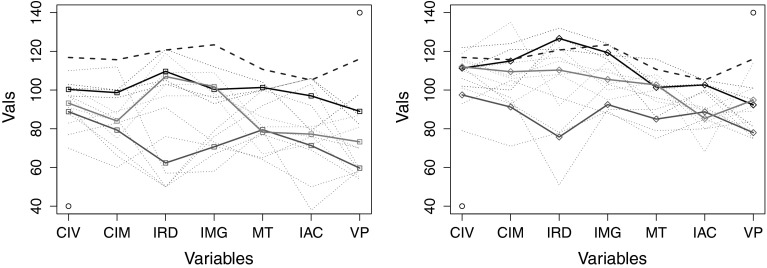
The line plots for clusters that were discovered by applying K-Means over (*left*) $t_{\rm pre }$ and (*right*) $t_{\rm post }$. The outliers were excluded from the data. The depicted clusters are discovered over the complete TBI data rather than bootstraped samples (they are used only for individual runs). The* bold lines* represent the centroids of the clusters, while* thin dotted lines* depict the patients. The colours show which clusters from $t_{\rm pre }$ and $t_{\rm post }$ are related to each other. (Color figure online)

**Table 5:** Meta Information on the clustering model from Fig. 7

Colour	Members	
	@ $t_{\rm pre }$	@ $t_{\rm post }$
Black	#6, #1, #8	#6, #3, #5
Red	#7, #9, #10, #14, #12, #13	#7, #9, #10, #14
Green	#2, #4, #3, #5	#2, #4, #1, #8, #12, #13

To reflect on the low accuracies of the label prediction, we show the clusters from $t_{\rm pre }$ and $t_{\rm post }$ in Fig. 7, after removing the outliers. The membership information is given in Table 5. We can observe how patients move closer to the controls (depicted as a dashed blue line) from $t_{\rm pre }$ to $t_{\rm post }$. The clusters take into account the changes in the similarity among patients, but this does not lead to meaningful predictions. Upon inspecting the dataset, we discovered that the ICP variable is not correlated with other attributes in the TBI dataset. One would expect this to be true, because the selected cognitive tests that are not correlated to each other. We can clearly see from the above experiments that it is not really possible to predict the ICP values from the values of other cognitive tests.

**Table 6:** Label prediction accuracies of each patient for EvoLabelPred with * GroundTruth* based on* ICP* ICP variable; PCA was applied over the TBI dataset prior to the learning of the evolutionary model

ID	PCA with * K* = 3	
	With outliers	Without outliers
#1	0.00	0.00
#2	97.73	93.06
#3	47.92	11.69
#4	100.00	100.00
#5	0.00	0.00
#6	0.00	0.00
#7	0.00	0.00
#8	98.10	88.57
#9	1.79	0.00
#10	92.31	91.57
#11	100.00	–
#12	93.81	90.41
#13	6.93	5.00
#14	59.78	10.71
#15	96.55	–

We conducted further experiments to test this non-correlation among the variables. We applied PCA on TBI dataset prior to model learning. We present the results in Table 6 with EvoLabelPred model based on* K* = 3 clusters and conditional probabilities-based label prediction. Although we see slight improvement compared to our results without PCA (cf. Table 4), the overall performance is low. After removing the outliers from the label prediction model, the performance of our label prediction even dropped considerably. This means that the ICP variable does not predict well whether the patient has recovered or not (contrary to the expectations).

## Conclusion

In this paper, we have investigated the problem of predicting the evolution of patients treated after brain injury, i.e. *predicting their recovery* and their *projection into the future*. We have proposed a mining workflow.

*Key points*: Our mining workflow, which consists of two individual methods, EvolutionPred and EvoLabelPred, clusters patients on similarity (of their assessments) before and after the treatment began, and then it tracks how each cluster evolves. It builds a cluster evolution graph that captures the transitions of patient clusters before (PRE) to after treatment (POST). Once the cluster evolution graph has been constructed, our methods EvolutionPred and EvoLabelPred use the clusters and their transitions to project each patient to a future moment, and predict their recovery label, respectively. The *projections* and *predictions* are done on the basis of what is known on the patients thus far.

We have experimentally validated our methods on the Trauma Brain Injury dataset [16]. We have first applied the EvolutionPred on known data and have shown that the projected values are almost identical to the true ones. Then, we have compared the projected assessments to those of a control population, and we have shown that some patient assessments are projected close to the controls. We studied treatment after brain trauma, but our EvolutionPred is applicable to any impairment, where progression or the process of recovery is of interest. The clusters we find may be of use in personalized medicine. Application of EvoLabelPred did not go as smooth. The models that we learned were predictive for only a part of the data. A major reason for this low performance was that the selected target variable was not sufficiently predictive on these data. We have to investigate this issue in the future, together with the medical experts.

*Shortcomings and future work*: The projected assessments have not yet been evaluated against the assertions of a human expert about the patients’ health state after treatment. We are currently in the process of acquiring such data for an additional evaluation. A further shortcoming is that we ignore the duration of treatment; this is planed as future step.

The evolution of brain trauma or impairment conditions is difficult to measure at the functional level. However, the scholars anticipate that the use of neuroimaging, e.g. MEG, could lead to the detection of progressive changes in the connectivity patterns even *before* they translate into changes at the memory, movement or orientation functions. Regularly recording MEG images before and during treatment of patients allows a more effective evaluation of treatment by providing hints and indicators about the effectiveness of a particular therapy. A next step for our work will be the integration of MEG data into our mining workflow to check whether the evolution of patients towards the subcohort of controls can be modelled more effectively with the MEG images.

## Footnotes

1 The one moment is “before” the treatment began, the other moment is “after” the treatment began, but without knowing when exactly the treatment began or ended.

2 The acronyms were derived from the original Spanish names. Therefore, the textual descriptions do not reflect the acronyms. We also provide the English acronyms in parentheses.

3 The experts considered ICP as more trustworthy than WNC.

4 We denote the centroid of an arbitrary cluster $clu$ as $\widehat{clu}$.

## References

[1] Cox D (1972). Regression models and life-tables. J R Stat Soc Ser B (Methodol).

[2] Fitzmaurice GM, Laird NM, Ware JH (2012). Applied longitudinal analysis.

[3] Marcano-Cedeño A, Chausa P, García A, Cáceres C, Tormos J, Gómez E (2013). Data mining applied to the cognitive rehabilitation of patients with acquired brain injury. J Expert Syst Appl.

[4] Rughani AI, Dumont TM, Lu Z, Bongard J, Horgan MA, Penar PL, Tranmer BI (2010). Use of an artificial neural network to predict head injury outcome: clinical article. J Neurosurg.

[5] Shi HY, Hwang SL, Lee KT, Lin CL (2013). In-hospital mortality after traumatic brain injury surgery: a nationwide population-based comparison of mortality predictors used in artificial neural network and logistic regression models: clinical article. J Neurosurg.

[6] Andrews PJ, Sleeman DH, Statham PF, McQuatt A, Corruble V, Jones PA, Howells TP, Macmillan CS (2002). Predicting recovery in patients suffering from traumatic brain injury by using admission variables and physiological data: a comparison between decision tree analysis and logistic regression. J Neurosurg.

[7] Brown A, Malec J, McClelland R, Diehl N, Englander J, Cifu D (2005). Clinical elements that predict outcome after traumatic brain injury: a prospective multicenter recursive partitioning (decision-tree) analysis. J Neurotrauma.

[8] Rovlias A, Kotsou S (2004). Classification and regression tree for prediction of outcome after severe head injury using simple clinical and laboratory variables. J Neurotrauma.

[9] Nagin DS, Odgers CL (2010). Group-based trajectory modeling in clinical research. Annu Rev Clin Psychol.

[10] Niyonkuru C, Wagner AK, Ozawa H, Amin K, Goyal A, Fabio A (2013). Group-based trajectory analysis applications for prognostic biomarker model development in severe TBI: a practical example. J Neurotrauma.

[11] Ram N, Grimm KJ (2009). Methods and measures: growth mixture modeling: a method for identifying differences in longitudinal change among unobserved groups. Int J Behav Dev.

[12] Stein NR, Dickstein BD, Schuster J, Litz BT, Resick PA (2012). Trajectories of response to treatment for posttraumatic stress disorder. Behav Ther.

[13] Xie H, Mayo N, Koski L (2011). Identifying and characterizing trajectories of cognitive change in older persons with mild cognitive impairment. Dement Geriatr Cogn Disord.

[14] Tucker A, Garway-Heath D (2010). The pseudotemporal bootstrap for predicting glaucoma from cross-sectional visual field data. IEEE Trans Inf Tech Biomed.

[15] Li Y, Swift S, Tucker A (2013). Modelling and analysing the dynamics of disease progression from cross-sectional studies. J Biomed Inform.

[16] Castellanos NP, Paul N, Ordonez VE, Deuynck O, Bajo R, Campo P, Bilbao A, Ortiz T, Pozo FdPdP, Maestu F (2010). Reorganization of functional connectivity as a correlate of cognitive recovery in acquired brain injury. Brain.

[17] Spiliopoulou M, Ntoutsi I, Theodoridis Y, Schult R (2006) MONIC—modeling and monitoring cluster transitions. In: Proc. of 12th ACM SIGKDD int. conf. on knowledge discovery and data mining (KDD’06). ACM, pp 706–711

[18] Ntoutsi I, Spiliopoulou M, Theodoridis Y (2011) Summarizing cluster evolution in dynamic environments. In: Int. conf. on computational science and Its applications, ICCSA 2011. pp 562–577

[19] Oliveira M, Gama J (2012). A framework to monitor clusters evolution applied to economy and finance problems. Intell Data Anal.

[20] Siddiqui ZF, Oliveira M, Gama J, Spiliopoulou M (2012) Where are we going? Predicting the evolution of individuals. In: Proceeding of the IDA 2012 conference on intelligent data analysis, vol. LNCS 7619. Springer, New York, pp 357–368

[21] Gaffney S, Smyth P (1999) Trajectory clustering with mixtures of regression models. In: 5th int. conf. on knowledge discovery and data mining. pp 63–72 . DOI10.1145/312129.312198

[22] Krempl G, Siddiqui ZF, Spiliopoulou M (2011). Online clustering of high-dimensional trajectories under concept drift.

[23] Siddiqui ZF, Krempl G, Spiliopoulou M, Pena JM, Paul N, Maestu F (2014) Are some brain injury patients improving more than others? In: The 2014 international conference on brain informatics and health (BIH ’14). Warsaw, Poland

[24] Ntoutsi E, Spiliopoulou M, Theodoridis Y (2014). FINGERPRINT—summarizing cluster evolution in dynamic environments. Int J Data Warehous Min.

[25] Hyndman RJ, Koehler AB (2006) Another look at measures of forecast accuracy. Int J Forecast 22(4):679–688. doi:10.1016/j.ijforecast.2006.03.001. http://www.sciencedirect.com/science/article/pii/S0169207006000239

[26] Breiman L (1996). Bagging predictors. Mach Learn.

